# Data on charge-transfer interaction between 1-methyl-3-trifluoromethyl-2-pyrazoline-5-one with PA, CLA, TFQ, DDQ and TCNQ π-acceptors

**DOI:** 10.1016/j.dib.2021.107137

**Published:** 2021-05-15

**Authors:** Abdel Majid A. Adam, Tariq A. Altalhi, Hosam A. Saad, Moamen S. Refat, Mohamed S. Hegab

**Affiliations:** a Department of Chemistry, College of Science, Taif University, P.O. Box 11099, Taif 21944, Saudi Arabia; b Deanship of Supportive Studies (D.S.S.), Taif University, P.O. Box 11099, Taif 21944, Saudi Arabia

**Keywords:** Charge-transfer interaction, Donor, Acceptor, Stoichiometry

## Abstract

This article is related to a research paper entitled “Exploring the charge-transfer chemistry of fluorine-containing pyrazolin-5-ones: The complexation of 1-methyl-3-trifluoromethyl-2-pyrazoline-5-one with five π-acceptors” [J. Mol. Liq. 331 (2021) 115814] [1]. Herein we present photographic data that showed the color change after mixing methanolic solutions of 1-methyl-3-trifluoromethyl-2-pyrazoline-5-one (donor) with each of the investigated π-acceptor [picric acid (PA), chloranilic acid (CLA), fluoranil (TFQ), DDQ, and TCNQ]. Stoichiometry data for the interaction of the donor with all acceptors determined in solution state by the spectrophotometric titration method and the Job's continuous variation method were presented. The data presented are useful for understand that the charge-transfer (CT) complexation between a donor and an acceptor, generally, is characterized by a strong color change, and to understand the stoichiometry between these molecules.

## Specifications Table

**Table:** 

Subject	Chemistry
Specific subject area	Charge-transfer (CT) dynamics
Type of data	ImageGraph/Plot
How data were acquired	HACH LANGE GmbH UV/VIS Spectrophotometer (Model DR6000 Benchtop), OriginPro 9 software
Data format	Raw and analysed
Parameters for data collection	All data were collected on CTCs generated in methanol at room temperature.
Description of data collection	Methanolic solutions of the donor and each acceptor were mixed and the resultant products were scanned using a UV/Vis spectrophotometer. The baseline was collected using the solvent only (methanol) before measuring the UV/Vis spectra of the solutions. The UV/Vis spectra dataset were provided as separated Excel sheet. These UV/Vis spectra were compared with those from the unreacted starting reagents alone to verify the stoichiometry of the interaction.
Data source location	Department of Chemistry, College of Science, Taif University, Taif, Saudi Arabia
Data accessibility	Data are available with the article.
Related research article	A.M.A. Adam, Tariq A. Altalhi, H.A. Saad, M.S. Refat, M.S. Hegab, Exploring the charge-transfer chemistry of fluorine-containing pyrazolin-5-ones: The complexation of 1-methyl-3-trifluoromethyl-2-pyrazoline-5-one with five π-acceptors, J. Mol. Liq. 331 (2021) 115814. https://doi.org/10.1016/j.molliq.2021.115814

## Value of the Data

• Pyrazole derivatives commonly used in medicinal chemistry. Exploring the charge-transfer (CT) properties of pyrazole derivatives may be useful toward improving their clinical efficacy and expanding the range of their medicinal applications.
• Determining the stoichiometry of the complexation between one of the pyrazole derivatives (namely 1-methyl-3-trifluoromethyl-2-pyrazoline-5-one) with different acceptors is particularly important to understand the mode of the interaction of these derivatives toward improving their uses and chemical, physical, and biological applications. These data are useful for researchers applying the CT interaction in the fields of chemistry, biochemistry, physics, biology, medicine, and pharmacology.
• The most important and useful approaches to verify the stoichiometry of the complexation between molecules are the spectrophotometric titration method and the Job's continuous variation method. These approaches are easily performed.

## 1 Data Description

**Fig. 1:**
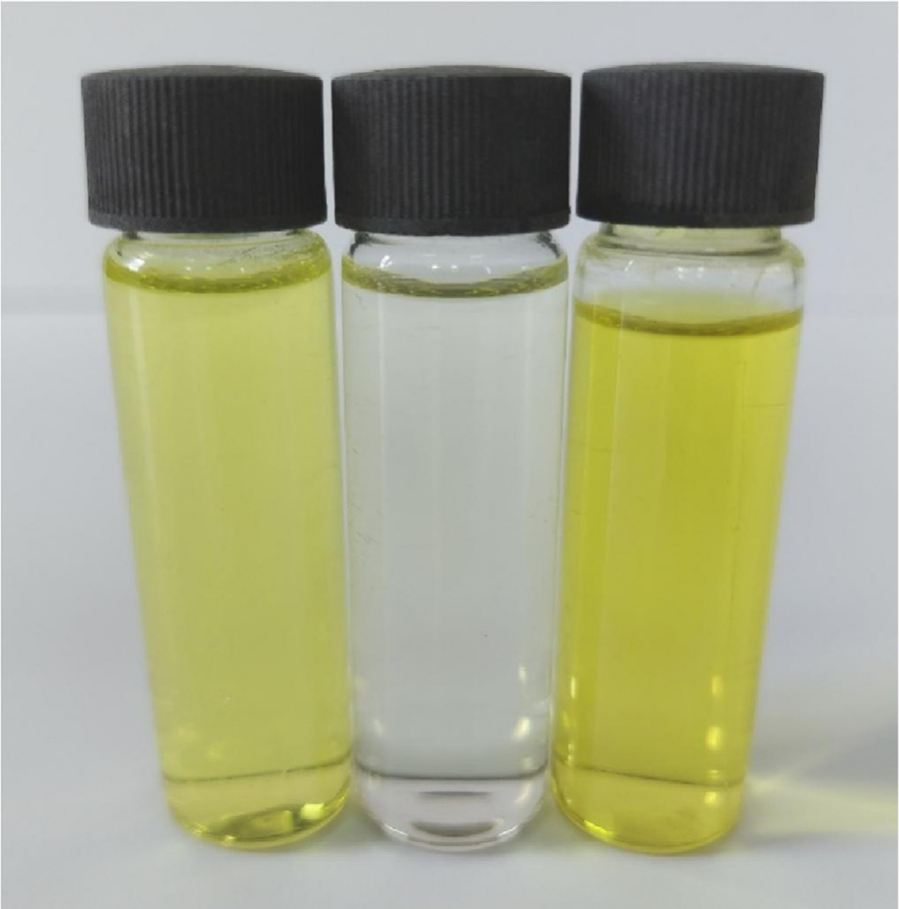
Color change upon mixing PA acceptor (*far left; yellow*) with the donor (*middle; colorless*) to generate the CT complex (*far right; instance yellow*). (For interpretation of the references to color in this figure legend, the reader is referred to the web version of this article.)

**Fig. 2:**
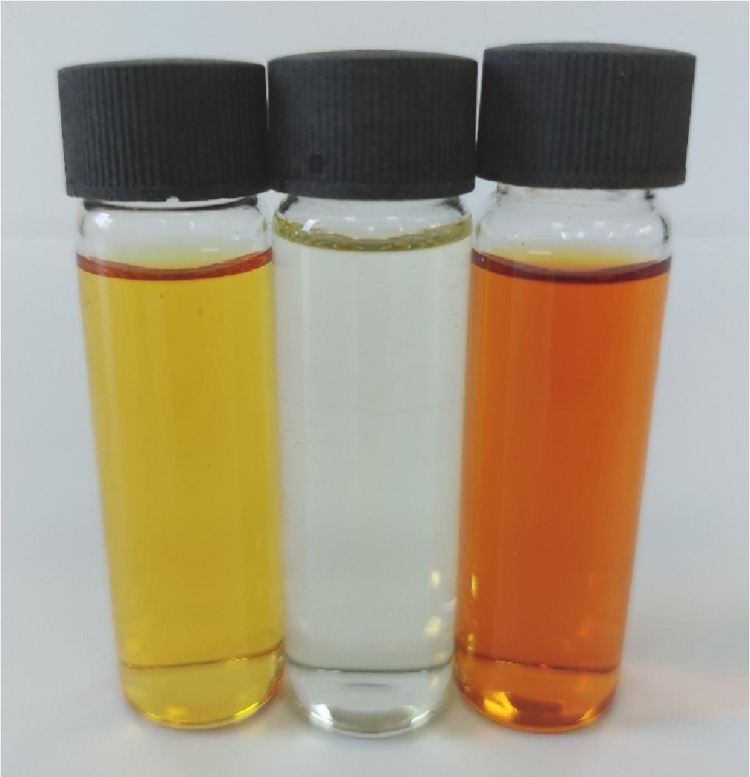
Color change upon mixing CLA acceptor (*far left; brown*) with the donor (*middle; colorless*) to generate the CT complex (*far right; intense brown*). (For interpretation of the references to color in this figure legend, the reader is referred to the web version of this article.)

**Fig. 3:**
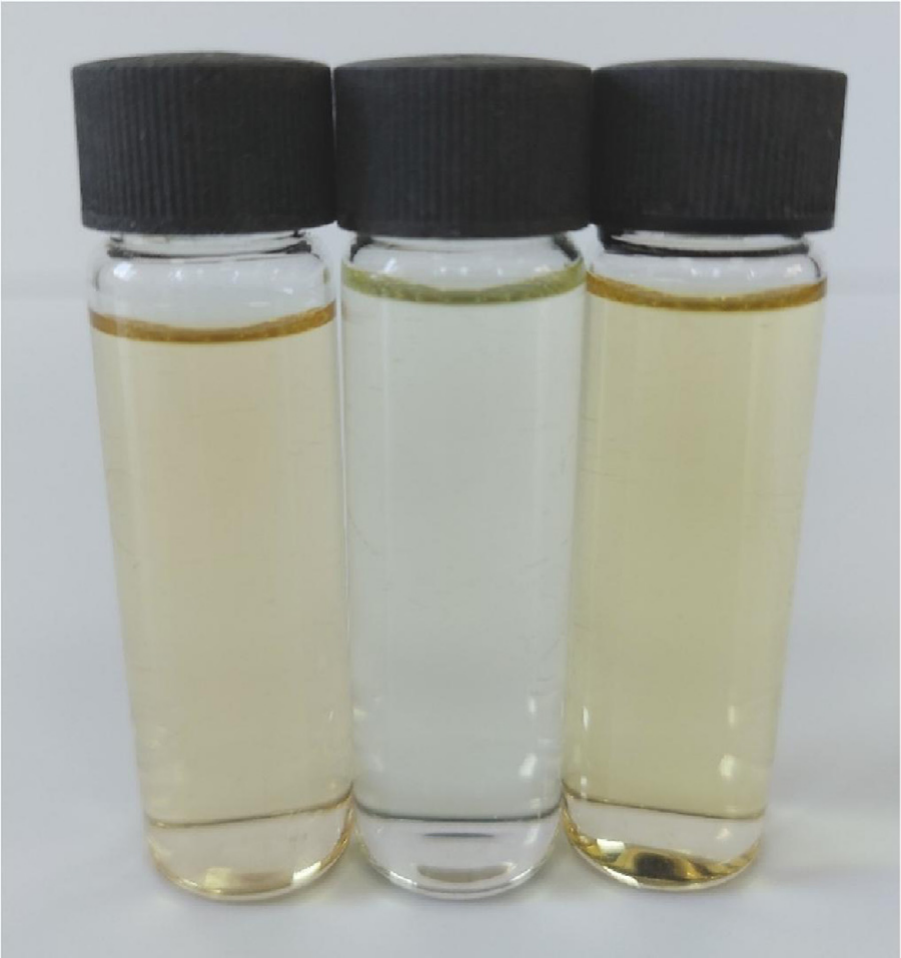
Color change upon mixing TFQ acceptor (*far left; pale brown*) with the donor (*middle; colorless*) to generate the CT complex (*far right; light brown*). (For interpretation of the references to color in this figure legend, the reader is referred to the web version of this article.)

**Fig. 4:**
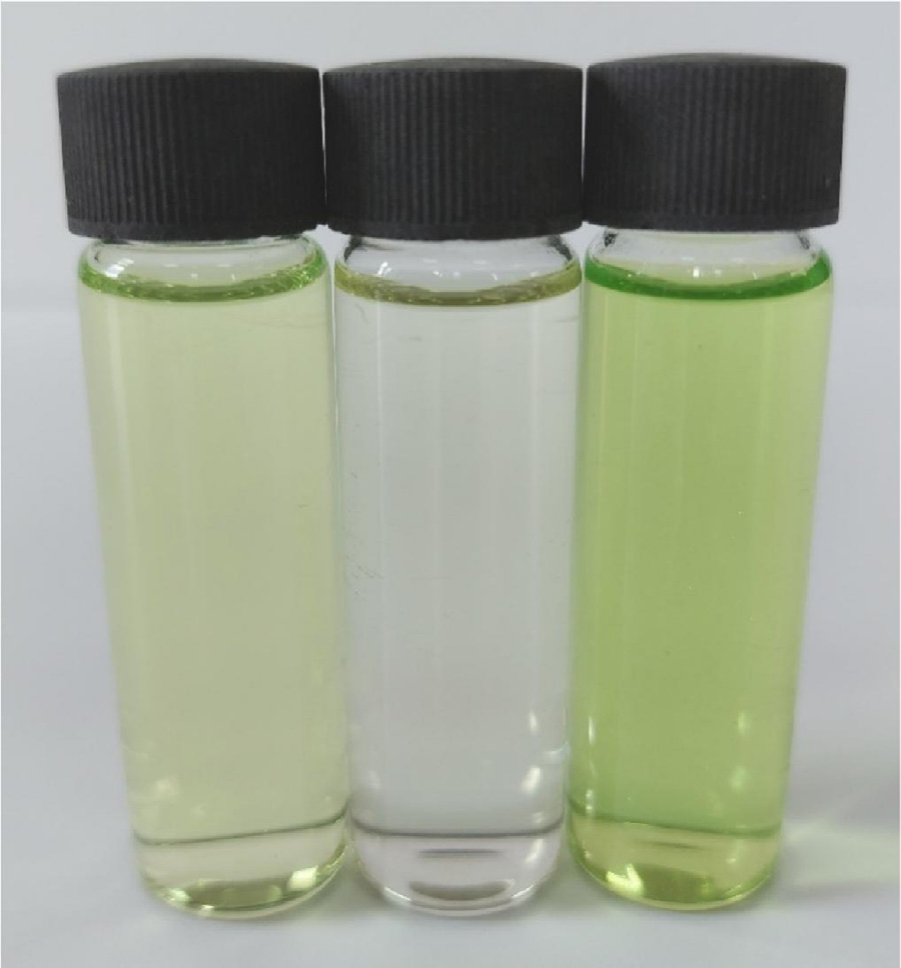
Color change upon mixing TCNQ acceptor (*far left; light green*) with the donor (*middle; colorless*) to generate the CT complex (*far right; intense green*). (For interpretation of the references to color in this figure legend, the reader is referred to the web version of this article.)

**Fig. 5:**
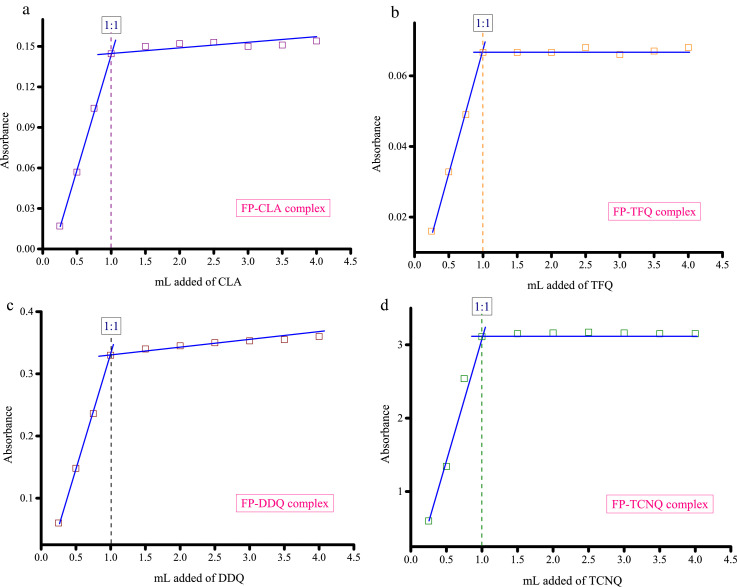
**a.** Stoichiometry of the interaction between the donor and CLA acceptor determined by spectrophotometric titration method (concentration of donor and CLA is 5 × 10^−4^ M). **b.** Stoichiometry of the interaction between the donor and TFQ acceptor determined by spectrophotometric titration method (concentration of donor and TFQ is 5 × 10^−4^ M). **c.** Stoichiometry of the interaction between the donor and DDQ acceptor determined by spectrophotometric titration method (concentration of donor and DDQ is 5 × 10^−4^ M). **d.** Stoichiometry of the interaction between the donor and TCNQ acceptor determined by spectrophotometric titration method (concentration of donor and TCNQ is 5 × 10^−4^ M).

**Fig. 6:**
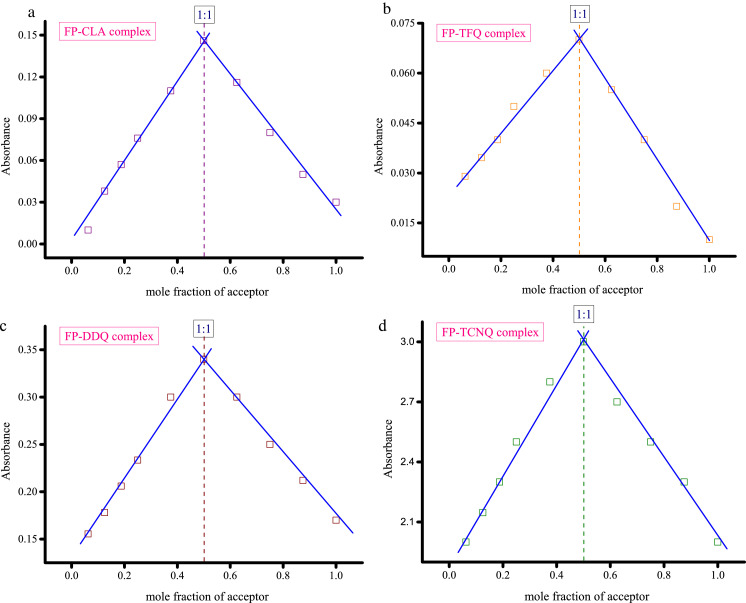
**a.** Stoichiometry of the interaction between the donor and CLA acceptor determined by Job's continuous variation method (concentration of donor and CLA is 5 × 10^−4^ M). **b.** Stoichiometry of the interaction between the donor and TFQ acceptor determined by Job's continuous variation method (concentration of donor and TFQ is 5 × 10^−4^ M). **c.** Stoichiometry of the interaction between the donor and DDQ acceptor determined by Job's continuous variation method (concentration of donor and DDQ is 5 × 10^−4^ M). **d.** Stoichiometry of the interaction between the donor and TCNQ acceptor determined by Job's continuous variation method (concentration of donor and TCNQ is 5 × 10^−4^ M).

Methanolic solutions of PA, CLA, TFQ, and TCNQ acceptors (1 × 10^−3^ M) were each individually mixed with the FP donor, also solubilized in methanol at the same concentration [2]. Striking color changes visible to the naked eye occurred as pictured in Fig. 1, Fig. 2, Fig. 3, Fig. 4. The complex formed between the donor and PA appeared intense yellow, CLA intense brown, TFQ light-brown, and TCNQ intense green. The UV/Vis spectra dataset for all complexes were provided as separated Excel sheet (Supplementary material). Fig. 5 (a–d) contains the curves generated for the donor-CLA, donor-TFQ, donor-DDQ, and donor-TCNQ systems using the spectrophotometric titration method [3] (the raw data associated with this figure were based on one measurement, and listed in Table 1), while the plots in Fig. 6 (a–d) present those obtained using Job's continuous variation method [4] (the raw data associated with this figure were based on one measurement, and listed in Table 2).

**Table 1:** Absorbance of the donor-CLA, donor-TFQ, donor-DDQ, and donor-TCNQ systems at different acceptor volume (the raw datasfor Fig. 5).

mL added of acceptor	Absorbance			
	CLA	TFQ	DDQ	TCNQ
0.25	0.017	0.016	0.06	0.6
0.5	0.0569	0.0328	0.148	1.342
0.75	0.1042	0.049	0.236	2.54
1	0.1445	0.0666	0.33	3.11
1.5	0.15	0.0666	0.34	3.15
2	0.152	0.0666	0.345	3.16
2.5	0.153	0.068	0.35	3.17
3	0.15	0.066	0.353	3.16
3.5	0.151	0.067	0.355	3.15
4	0.154	0.068	0.36	3.15

**Table 2:** Absorbance of the donor-CLA, donor-TFQ, donor-DDQ, and donor-TCNQ systems at different mole fraction of acceptor (the raw data for Fig. 6).

Mole fraction of acceptor	Absorbance			
	CLA	TFQ	DDQ	TCNQ
0.0625	0.01	0.029	0.1555	2
0.125	0.038	0.0346	0.178	2.147
0.1875	0.057	0.04	0.206	2.3
0.25	0.076	0.05	0.2335	2.5
0.375	0.11	0.06	0.3	2.8
0.5	0.146	0.07	0.34	3
0.625	0.116	0.055	0.3	2.7
0.75	0.08	0.04	0.25	2.5
0.875	0.05	0.02	0.212	2.3
1	0.03	0.01	0.17	2

## 2 Experimental Design, Materials and Methods

### 2.1 Materials

Analytical-grade TCNQ (C_12_H_4_N_4_; 204.19 g/mol; purity 98%), DDQ (C_8_Cl_2_N_2_O_2_; 227.00 g/mol; purity 98%), TFQ (C_6_F_4_O_2_; 180.06 g/mol; purity 97%), CLA (C_6_H_2_Cl_2_O_4_; 208.98 g/mol; purity ≥ 98%), PA (C_6_H_3_N_3_O_7_; 229.10 g/mol; purity ≥ 98%), and 1-methyl-3-trifluoromethyl-2-pyrazoline-5-one (donor) (C_5_H_5_F_3_N_2_O; 166.1 g/mol; purity 95%) were obtained from Merck KGaA (Darmstadt, Germany) and UFC Biotechnology (Amherst, NY, USA).

### 2.2 Methods

1. Donor and acceptor solutions were individually prepared at 5 × 10^−4^ M in methanol in 25-mL volumetric flasks.
2. The solubilized donor (1 mL) was combined with each solubilized acceptor (1 mL) and methanol (3 mL) in 5-mL glass tubes to generate the donor-PA, donor-CLA, donor-TFQ, donor-DDQ, and donor-TCNQ systems.
3. The UV-visible spectra of the free donor, free acceptors, and synthesized complexes were collected at room temperature from 200 to 800 nm using a Perkin-Elmer Lambda 25 UV/Vis spectrophotometer and used to determine the CT band (λ_CT_) for each system.
4. To verify the stoichiometry of the interaction between the donor and each of the acceptors using the spectrophotometric titration method, the absorbances (λ_CT_) of 10 standard solutions with varied donor to acceptor molar ratios (from 4:1 to 1:4) were plotted against the volume of the acceptor in each standard solution.
5. To verify the stoichiometry of the interaction between the donor and each acceptor using Job's continuous variation method, the absorbances (λ_CT_) of 10 standard solutions with varied molar fractions of donor and acceptor (C_donor_ + C_acceptor_) were plotted against the molar fraction of the acceptor in each standard solution.

## CRediT Author Statement

**Abdel Majid A. Adam:** Data curation, Writing – original draft, Writing – review & editing; **Tariq A. Altalhi:** Conceptualization, Methodology, Software, Supervision; **Hosam A. Saad:** Visualization, Investigation, Software, Validation; **Moamen S. Refat:** Conceptualization, Methodology, Software, Supervision; **Mohamed S. Hegab:** Visualization, Investigation, Software, Validation.

## Declaration of Competing Interest

The authors declare that they have no known competing financial interests or personal relationships that could be perceived to have influenced the work reported in this article.

## Appendix Supplementary materials

Image, application 1
